# Ecosystem Alterations and Species Range Shifts: An Atlantic-Mediterranean Cephalaspidean Gastropod in an Inland Egyptian Lake

**DOI:** 10.1371/journal.pone.0156760

**Published:** 2016-06-01

**Authors:** Edwin Cruz-Rivera, Manuel António E. Malaquias

**Affiliations:** 1 Department of Biological Sciences, University of the Virgin Islands, #2 John Brewers Bay, St. Thomas, 00802, United States Virgin Islands; 2 Phylogenetic Systematics and Evolution Research Group, Section of Taxonomy and Evolution, Department of Natural History, University Museum of Bergen, University of Bergen, Bergen, PB 7800, 5020-Bergen, Norway; Naturhistoriska riksmuseet, SWEDEN

## Abstract

The eastern Atlantic and Mediterranean marine Cephalaspidea gastropod *Haminoea orbignyana* was collected from Lake Qarun (Fayoum, Egypt), a landlocked lake that has undergone a shift from freshwater to estuarine conditions in the past 100 years. Species identity was confirmed by both morphological (anatomical dissection and scanning electron microscopy) and molecular methods (COI gene phylogeny). Observations suggested a robust population of *H*. *orbignyana* in the lake with a density of *ca*. 64 individuals/m^2^ and *ca*. 105 egg masses/m^2^ during surveys conducted in the summer of 2013. The vast majority of snails and egg masses were found under rocks. Observations of egg masses in the lab showed a gradual change from whitish to yellow-green as the eggs matured and the release of veliger larvae alone after about a week. Although adult cephalaspideans readily consumed filamentous red and green algae, and cyanobacteria, laboratory trials showed that they consumed significantly more of the red alga *Ceramium* sp., than of the green alga *Cladophora glomerata*, with consumption of *Oscillatoria margaritifera* being similar to those on the two algae. When grown on these resources for 16 days, *H*. *orbignyana* maintained their mass on the rhodophyte and cyanobacterium, but not in starvation controls. No cephalaspideans grew over the course of this experiment. Lake Qarun has been periodically restocked with Mediterranean fishes and prawns since the 1920s to maintain local fisheries, which represents a possible route of colonization for *H*. *orbignyana*. Yet, based on literature records, it seems more likely that invasion of the lake by this gastropod species has occurred only within the last 20 years. As human activities redistribute species through direct and indirect means, the structure of the community of this inland lake has become unpredictable and the long-term effects of these recent introductions are unknown.

## Introduction

The literature on species invasions is largely focused on organisms that have altered significantly the structure of the communities in which they have been introduced [1–4]. Much less attention has been paid to species that have become established in new areas without noticeable effects on the environment, or to small species that are difficult to distinguish from local ones [2, 5, 6]. Incipient invasions, however, can have strong influence on the novel ecosystem over time, as the newly introduced species increases population size. For many small marine invertebrates that require microdissection or DNA data for positive identification, species introductions may not be noticed, sometimes until almost complete replacement of natives has occurred [2, 6–8]. Yet, it is such organisms that are often carried inadvertently by human activities to new habitats as adults or larvae. A lack of expert taxonomists for many of these animals, a phenomenon known as the *taxonomic impediment*, has compounded the problem of identifying small introduced species even in instances where they become locally dominant [5, 8].

Such is the case for cephalaspideans or bubble snails. Some tropical species may have unique color patterns that can be readily identified, but for many others it is unclear if the differences in coloration correspond to phenotypic differences or species-level traits [9, 10]. Shell morphology often only informs about higher taxonomic ranks (e.g. genera) and microdissection of the radula, gizzard plates, and reproductive system are required to establish species identity [7, 11–14]. In fact, these difficulties have previously resulted in the mistaken identification of invasive cephalaspideans and the wrong assignment of DNA sequences [6–8].

Human transport of species is only one dimension of the successful range expansion of many organisms. While propagules of a species can reach new habitats by either natural or human-mediated dispersal, their establishment in a new area requires reaching a suitable environment. In this regard, human alteration of ecosystems has increased the opportunities for invasion in many places that historically would have not allowed establishment of organisms now found there [15–17]. In the case of aquatic systems, eutrophication and salinization of freshwater habitats can alter water quality enough to compromise the stability of local aquatic flora and fauna [18–22], and favor the establishment of invaders [22–26]. Unregulated water use and salinization can transform a freshwater environment into a brackish, or even marine, system over a few decades [21, 22, 27].

**Fig 1:**
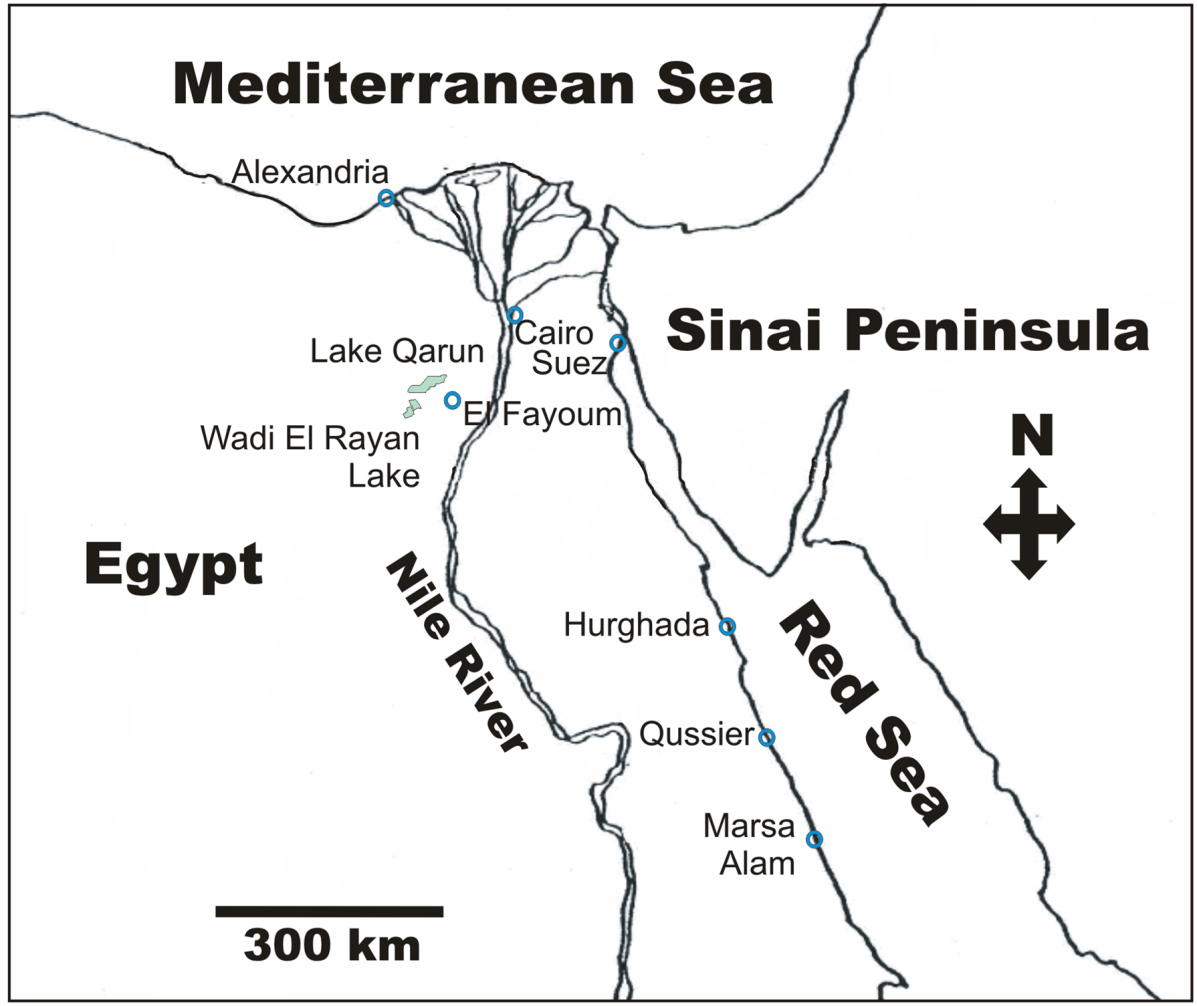
Location of Lake Qarun, El Fayoum, Egypt. The general location of the lake in relation to major cities and features is illustrated. The lake is located in the Fayoum depression.

Lake Qarun is a shallow inland water reservoir located in the Fayoum (= Fayum, Faiyum) depression of Egypt (Fig 1). With an average depth of 4 m, and dimensions of ca. 6 x 40 Km, the lake is the remnant of what constituted the freshwater Lake Moeries during pharaonic times. This original lake formed during the Pleistocene and remained a freshwater reservoir until circa 1550 AD [28–31]. Over the past several decades, development has changed the lands surrounding the lake considerably. Currently, water coming from the Nile is largely used for irrigation of agricultural fields and fish farms [32]. In addition, the construction of the Aswan High Dam in the 1960s significantly reduced flow of the small tributary of the Nile River that once fed the lake and stopped the seasonal Nile floods into the Fayoum [30]. Presently, the lake serves largely as drainage for the surrounding farmlands. There is no major outflow to the lake and the main route of water loss is evaporation [29, 33, 34]. The combination of salt runoff from fertilized soils, the decreased volume of freshwater flowing into the lake, and the high rates of evaporation, have contributed to a progressive salinization of this body of water over the past centuries, especially during the last 100 years [29, 31, 33–35]. According to modern measurements [29], the range of salinity in Lake Qarun fluctuates from a low of 27.1‰ in the spring to a high of 38‰ in the summer.

Not surprisingly, the fauna and flora of this lake have changed dramatically over time [28, 30, 31, 36–38]. This has been compounded by the restocking of the lake with Mediterranean fishes and prawns since 1928 to support local consumption [32, 39–42]. As a result, a progressive turnover of species has ensued, from freshwater to estuarine, and various introduced invertebrate species have now established populations in Lake Qarun. Data on molluscs include both species lists and studies on benthic population density and biomass [30, 32, 36, 37, 43–45]. These demonstrate a changing molluscan fauna over the decades when dominant freshwater species have been replaced successively by estuarine and marine ones [30, 37, 44].

During studies about the diversity and ecology of the invertebrates of Lake Qarun carried out between 2010 and 2013, snails of the marine cephalaspidean gastropod genus *Haminoea* were consistently found in collected benthic algae. No previous records of this genus are known from any completely landlocked lakes and historical accounts suggest that the animals are not native to this environment [30, 32, 45]. Due to the history of species replacements in this lake an investigation was set up in order to confirm the taxonomic identity of the snails and to gather knowledge about basic aspects of their biology such as abundances and trophic interactions in this unusual inland system. These data can be used to establish a baseline against which to compare the long-term effects of this introduced species.

## Materials and Methods

### Snail and egg mass abundances

Field access and collection of snails and algae were permitted by the administration of the Panorama Hotel, Lake Qarun, Egypt. No threatened or endangered species were collected or disturbed. Snails were obtained by picking up algae and rocks on the southern shore of Lake Qarun, Fayoum, Egypt (29.4840° N– 30.6545° E), near the hotel. This area was visited between 2010 and 2013 as part of various educational activities related to courses taught at the American University in Cairo. Algal collections and epifaunal surveys during these courses always revealed the presence of the same cephalaspidean haminoid gastropod. Occasionally, egg masses which resembled those of haminoids also came with the samples (see [11]). Although the first observations of the animals were done using surface transects, later assessments suggested that both the animals and their putative eggs were more abundant under rocks than above them. To test this, 0.5 m^2^ quadrats (n = 6) were used during June 2013. The quadrats were randomly placed along the water edge and submerged rocks within them were picked up to count snails and egg masses on the top or bottom (facing sediments) of the rock. These field population counts were conducted between 10:00 am and 12:00 pm. Because data could not be normalized by transformations, densities above and below rocks were compared using Mann-Whitney U tests [46].

### Identification of egg masses and embryonic development

Cephalaspideans were also collected and kept with algae in aerated tubs to confirm the identity of the egg masses and determine the type of larva produced [47–49]. Once laid, the egg masses were compared with those observed in the field (e.g. shape, size, color) and incubated at room temperature (ca. 24°C; salinity 32‰; pH 8.7) to observe embryonic development. Coarsely filtered water from Lake Qarun was renewed every 2–3 days in five Petri dishes containing a single egg mass. Development was followed until the emergence of larvae by observing the egg masses once a day (approximately at noon) under a dissecting microscope.

### Morphological and molecular taxonomic identification

Based on images of live animals taken in the field, external coloration and characters of the shell, the specimens were preliminarily identified as belonging to the species *Haminoea orbignyana*. In order to confirm the taxonomic status of these animals, several specimens were brought alive to the laboratory and frozen at -70°C before preserving them in 90% ethanol for morphological and DNA analyses. Three specimens were dissected and two of those were sequenced for the mitochondrial gene cytochrome *c* oxidase subunit I (COI). The cephalic shield was opened axially and the reproductive system, buccal bulb, and gizzard were removed. The three gizzard plates were extracted from inside the gizzard and, together with the radula, were cleaned with commercial bleach diluted to approximately 20% to remove soft tissue. The male reproductive system was first drawn with the aid of a drawing tube and then critical-point dried. All structures were mounted in metallic stubs for scanning electron microscopy (SEM) and coated with gold–palladium. Shells were cleaned by immersion in commercial bleach (diluted to approximately 20%) until all soft tissue have been digested and were photographed with an automontage stereomicroscope system. All hard and soft structures were compared with those from other Atlantic and Mediterranean species of *Haminoea* based on the diagnostic characters described by Malaquias and Cervera [11].

In order to produce a COI gene phylogeny, pieces of the foot from two of the dissected specimens were cut off and used to extract DNA. Extraction, amplification, and sequencing methods were as described by Eilertsen and Malaquias [12]. Sequences were verified by both forward and reverse comparisons, and assembled and edited using Sequencher 5.01 (Gene Codes Corp.). GenBank accession numbers for these sequences are KT339765 and KT339766. Twenty-nine sequences of worldwide species of *Haminoea* were downloaded from GenBank and aligned together with our two novel sequences using Muscle implemented in Geneious v8.0.3 (Biomatters Ltd). The best-fit model of evolution was chosen using jModeltest v.2.1.1 [50]. Phylogenetic analysis was conducted in MrBayes 3.1.2b [51, 52] with default priors for ten million generations, with a sampling frequency of 100 and three separate runs to ensure that independent analyses were converging on the same tree. Convergence of runs was diagnosed using the program Tracer 1.4 [53]. For each analysis the first 10000 trees were discarded (‘burn-in’ period = 10%). One member of the family Bullidae (*Bulla striata* Bruguière, 1792; GenBank Accession No DQ986564) was chosen as an outgroup taxon. Genetic distances were calculated in Mega 5.1 [54] by plotting pairwise uncorrected *p*-distances against total distances (transversions + transitions).

### Assessment of feeding habits

**Fig 2:**
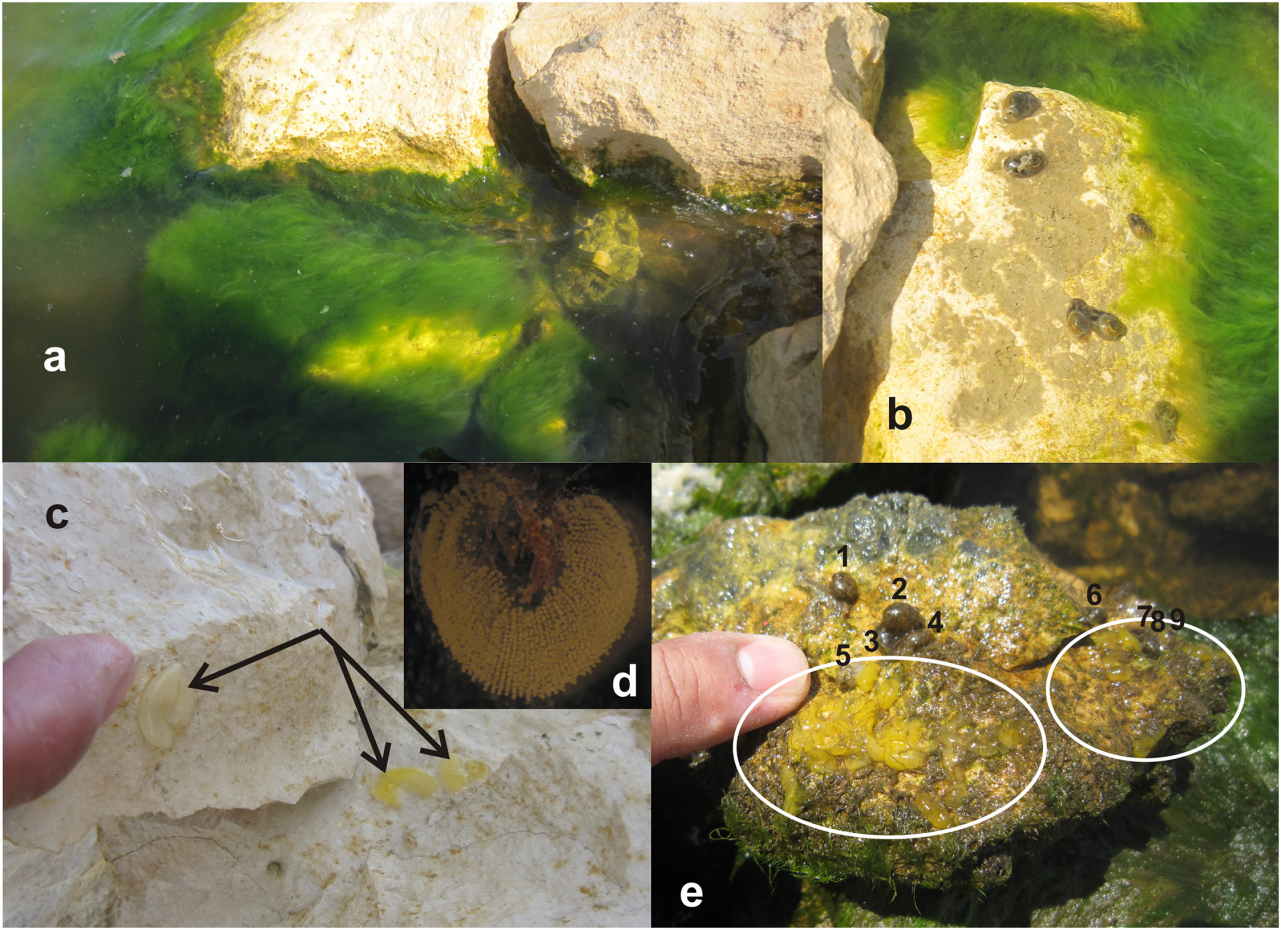
Field observations of *Haminoea orbignyana*. (A) Typical microhabitat from Lake Qarun where *H*. *orbignyana* were surveyed and collected showing a dense growth of *Cladophora glomerata* and (B) several individuals crawling over a rock. (C) Egg masses change in color from a milky white to a yellow-green as they mature (pointed by the arrows). (D) A close up (20x) of an egg mass collected from a mat of the red alga *Ceramium* sp. (red filaments at top and center). (E) The underside of a small rock showing nine *H*. *orbignyana* (numbered) and two clusters of egg masses (inside ovals).

The most visible primary producers where snails were abundant (Fig 2A and 2B) were *Cladophora glomerata* (Chlorophyta), *Ceramium* sp., (Rhodophyta) and *Oscillatoria margaritifera* (Cyanobacteria). No rhodophytes have been previously identified in the most recent floral surveys of Lake Qarun [55]. We quantified snail consumption of these foods by confining animals to a single diet and measuring the algal or cyanobacterial mass lost over time. Algal and cyanobacterial pieces were gently blotted dry to obtain initial masses. Plastic containers (N = 11) received a piece of alga or cyanobacterium, and either three adult cephalaspidean snails or no animals at all. Cups with algae, but no consumers, served as controls for changes in mass of diets unrelated to consumption (i.e. autogenic changes in mass [56, 57]). After allowing the snails to eat for two days, the foods were removed, blotted dry, and weighed again. Food consumption was calculated after correcting for autogenic changes in mass using the equation [(*H*_*o*_ x *C*_*f*_/*C*_*o*_)–*H*_*f*_]; where *H*_*o*_ and *H*_*f*_ represented initial and final wet masses of the food portions exposed to consumers, and *C*_*o*_ and *C*_*f*_ were the initial and final masses of the paired controls for autogenic changes.

We also compared survivorship and growth of *H*. *orbingyana* when fed *C*. *glomerata*, *Ceramium* sp., or *O*. *margaritifera*. For this, field-collected snails (ca. 5–8 mm shell length; 33.8 ± 1.42 mg) were individually placed in 120-mL plastic cups (N = 13) and assigned to either one of these three diets or a starvation control. Animals were gently dried with absorbent paper and weighed at the start of the assay, fed in excess (or starved), and re-weighed after 16 days to determine mass changes. Survivorship was checked daily and water was replaced every 2–3 days. It should be noted that some difficulties arose while maintaining animals on the cyanobacterial diet which constrained our ability to feed the animals *ad libitum* at times (see below). At the end of the experiment, survivorship was analyzed using a chi-square analysis. Growth was compared among treatments using a non-parametric Kruskal-Wallis test and Tukey-type pair-wise comparisons, after normality could not be corrected through any data transformations [46].

## Results

### Snail and egg mass abundances

**Fig 3:**
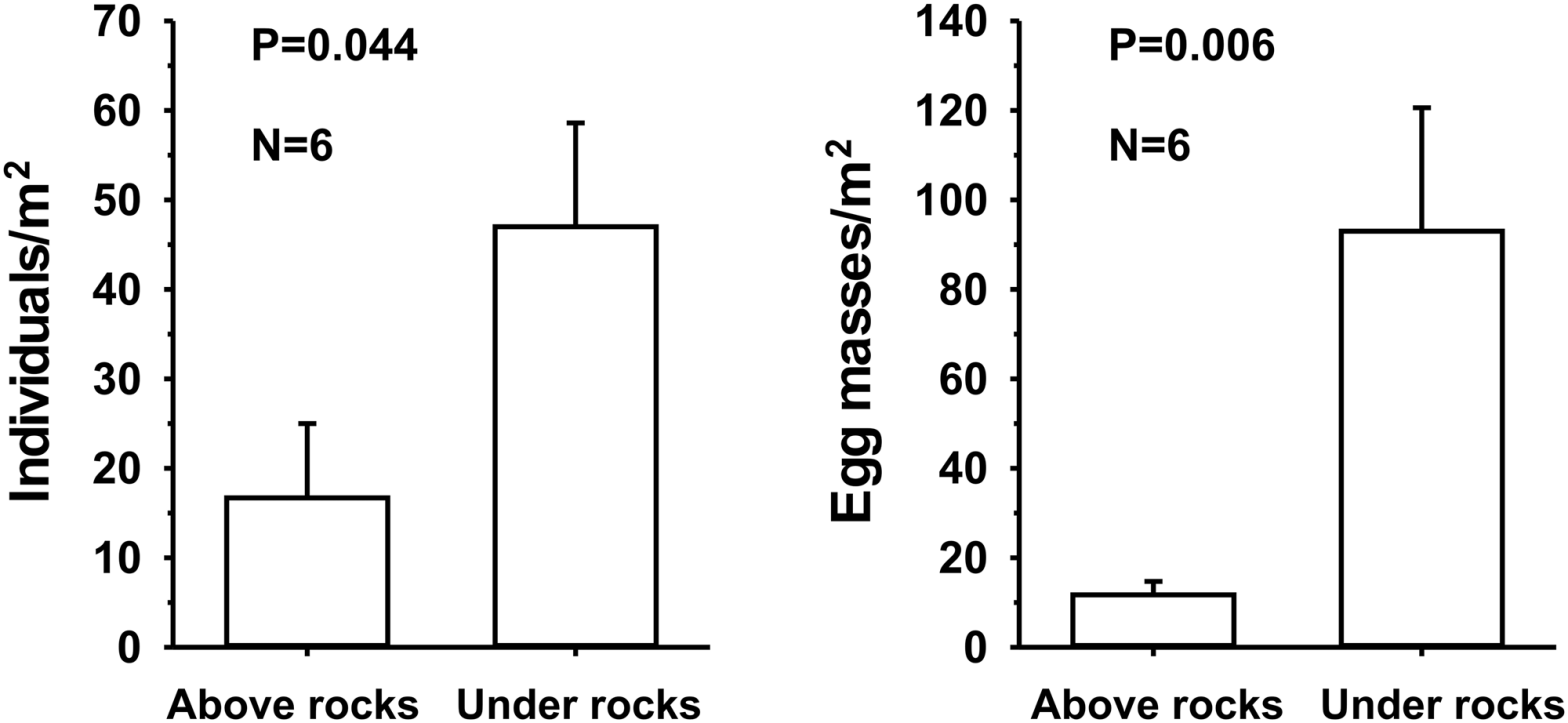
Field survey of *Haminoea orbignyana*. Densities of individuals and egg masses, above and below rocks, from the Lake Qarun southern shore. Bars represent means + 1 SE. P values are from Mann-Whitney U tests.

Animals and egg masses were considerably more abundant under rocks than above them (Figs 1 and 2). While mean density of snails was 16.7 ± 8.3 individuals/m^2^ above rocks, mean density under rocks was 47 ± 11.6 individuals/m^2^. This difference was statistically significant (P = 0.044, Mann-Whitney U test; Fig 3). Similarly, the density of egg masses above rocks (11.7 ± 3.0 masses/m^2^) was substantially and significantly lower than under rocks (93 ± 27.6 masses/m^2^) (P = 0.006, Mann-Whitney U test; Fig 3).

### Identification of egg masses and embryonic development

Egg masses developed readily in the lab until releasing planktonic veliger larvae about seven days after egg mass production. No direct development (i.e., crawling larvae) was observed. It was noted that recently laid egg masses were milky white but turned yellow-green as they developed (Fig 2C, 2D and 2E).

### Morphological and molecular taxonomic identification

**Fig 4:**
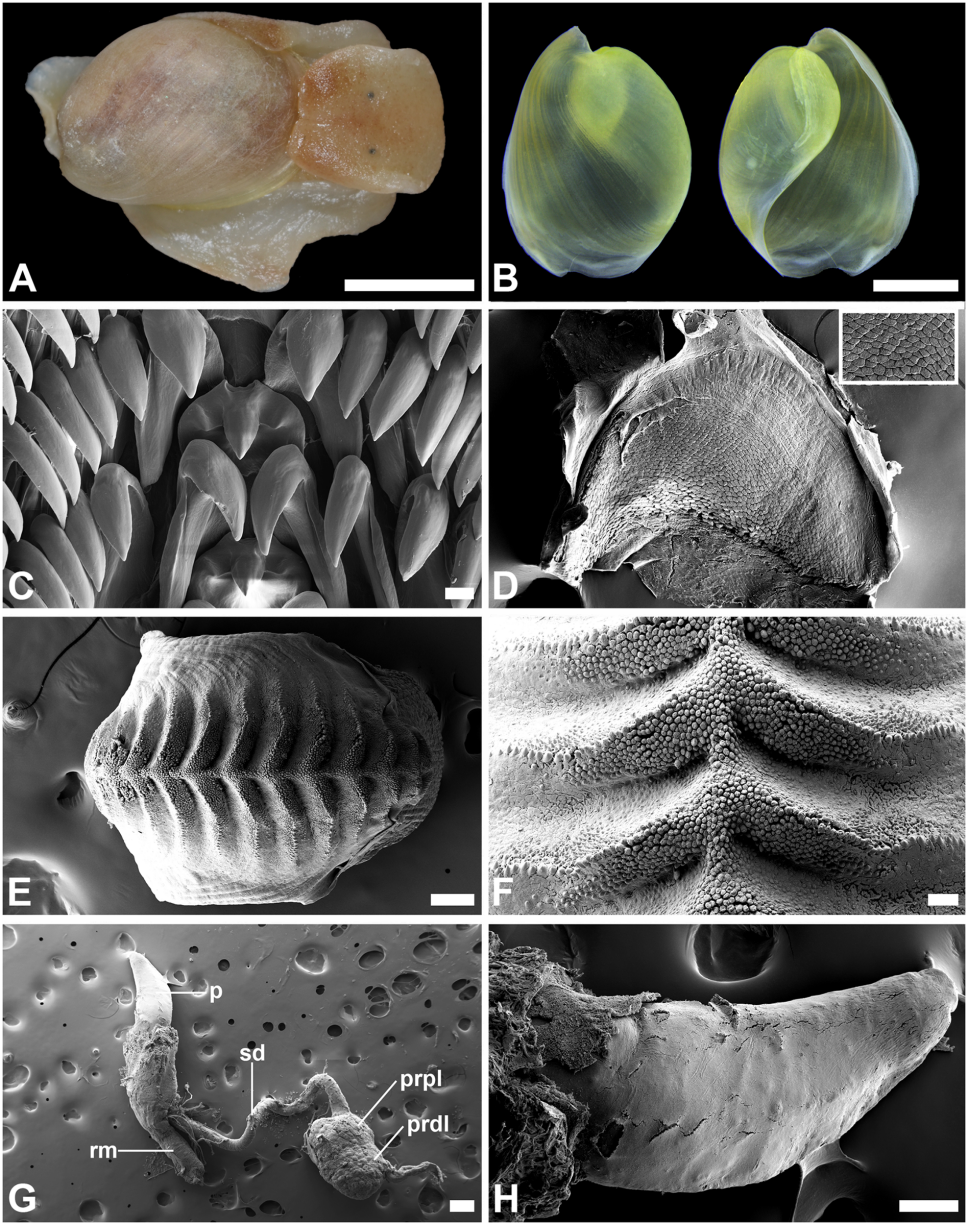
Anatomical details of *H*. *orbignyana* from Lake Qarun, Egypt (ZMBN 99936). (A) whole animal fixed in 90% EtOH (shell length = 6 mm); (B) adpertural (left) and apertural (right) views of shell; (C) radula with detail of raquidian, inner lateral and outer lateral teeth; (D) overall shape of jaw. Inset detail of the microsculpture; (E) dorsal view of gizzard plate; (F) detail of microsculpture of the gizzard plate taken from the mid-dorsal region; (G) male reproductive system (p, penis; prpl, prostate proximal lobe; prdl, prostate distal lobe; rm, retractor muscle; sd, seminal duct); (H) penis. Scale bars: A, 3 mm; B, 2 mm; C, 10 μm; D, 100 μm; E, 100 μm; F, 20 μm; G, 200 μm; H, 100 μm.

The morphological study of the three specimens dissected (voucher number: ZMBN 99936; University Museum of Bergen, Norway; Fig 4) confirmed our preliminary identification of the haminoid snails from Lake Qarun as belonging to the species *Haminoea orbignyana*. This is the only species in the eastern Atlantic Ocean and Mediterranean Sea to have a pear-shaped shell and, cumulatively, a smooth penial papilla and a prostate made up of two contiguous lobes. Moreover, the symmetrical jaws, smooth inner lateral teeth, and shape and distribution of rods along the active surface of the gizzard plates match known features of this species ([11]; Fig 4).

**Fig 5:**
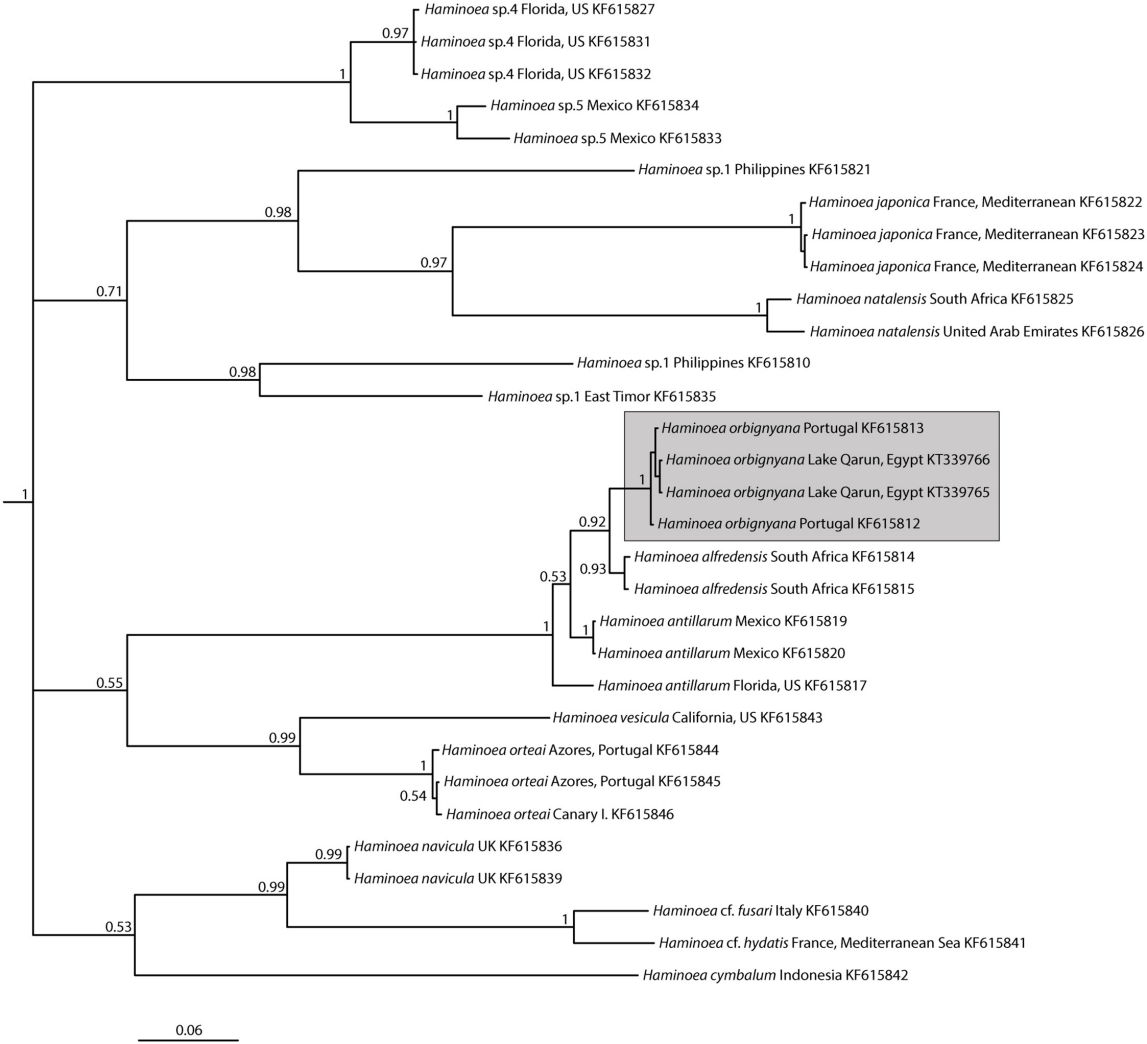
Genetic identification of lake haminoids. Phylogram generated by Bayesian inference analysis of COI gene sequences in MrBayes. Labels on branches represent node support as posterior probabilities (PPs). GenBank accession numbers are included after the geographical localities in the species names. Sequences attributed to the species *Haminoea orbignyana* are highlighted by a grey-dashed box. Outgroup removed for clarity.

The COI phylogenetic analysis included representatives of nine Atlantic, one East Pacific, and six Indo-West Pacific species of *Haminoea*, plus the western Pacific *Haminoea japonica*, an invasive species broadly distributed in the Mediterranean Sea (Fig 5). Aligned sequences were trimmed to a total of 680 bp and the best evolutionary model selected was the TVM + I + G with p-inv = 0.6080 and gamma shape = 1.4280. The two sequences from Lake Qarun, Egypt clustered together with those of *Haminoea orbignyana* from Portugal with a genetic distance of 0.000% (uncorrected *p*-distance).

### Assessment of food habits

**Fig 6:**
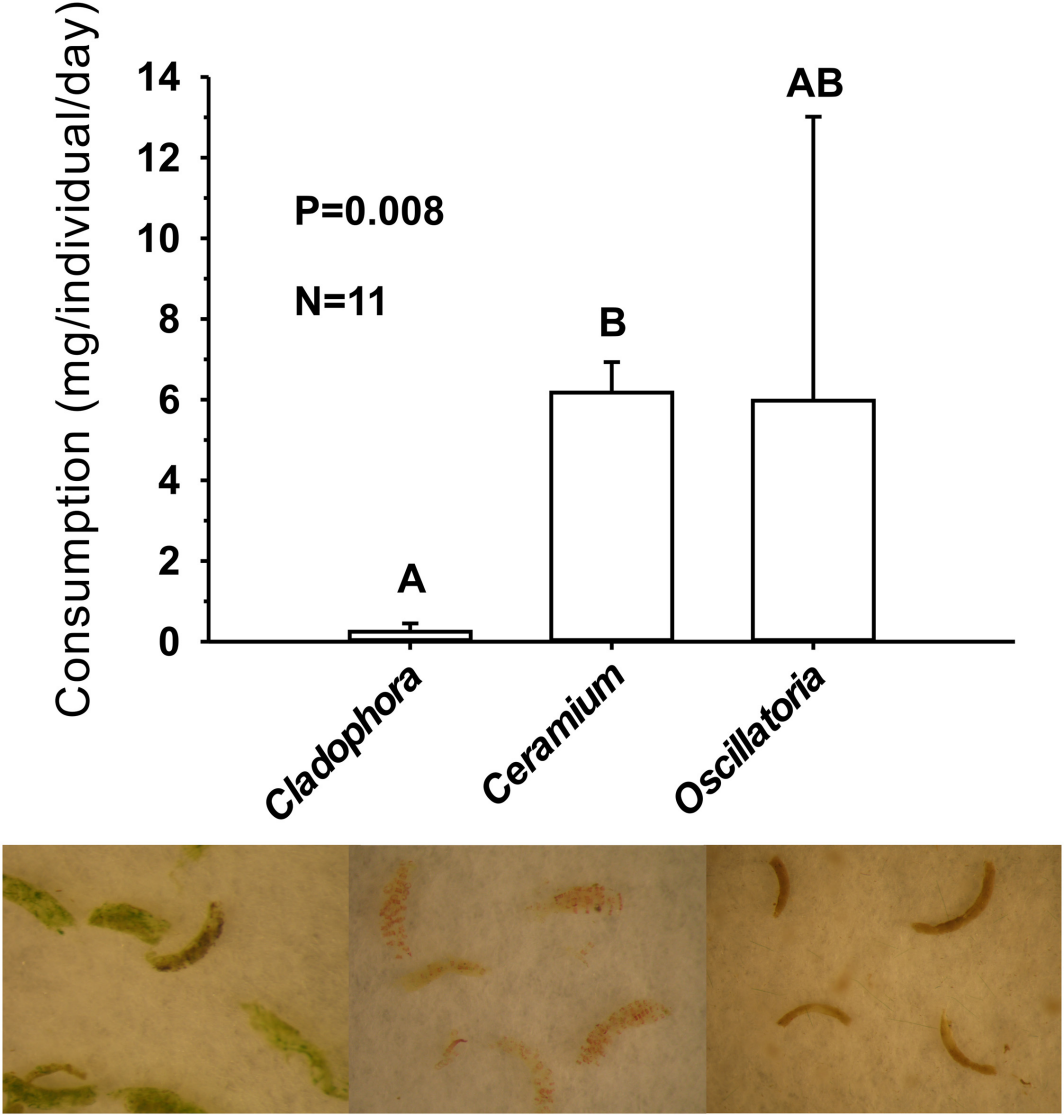
Cephalaspidean consumption of lake algae and cyanobacteria in no-choice conditions. Top: Feeding rates of *H*. *orbignyana* fed either on *Cladophora glomerata*, *Ceramium* sp., or *Oscillatoria margaritifera*. Bars represent means + 1 SE. The P value is from a Kruskal-Wallis test and significant groupings (letters above bars) are from Tukey-type *post hoc* tests. Bottom: Pictures of fecal pellets from this experiment showing the remains of ingested cells (40–42 x). The background of the pictures is a piece of filter paper for contrast. Notice the partially digested cells in some of the diets.

Animals readily consumed algae and cyanobacteria in the laboratory as indicated by direct observations under a dissecting microscope and the production of fecal matter containing food remnants (Fig 6). A statistically significant difference (P = 0.008, Kruskal-Wallis test) was found when consumption of the green alga *Cladophora* was compared to that of the red alga *Ceramium*, but variance in consumption of the cyanobacterium *Oscillatoria* was very high and statistically equivalent to that of the other two diets. This high variance most likely resulted from difficulties in weighing accurately loose filamentous cyanobacterial mats that grow over sediments. Because *Oscillatoria* filaments are motile, mats spread out in a few hours and formed very thin films that were difficult to harvest from the containers. As the filaments moved they also lost silt and sand grains that were part of the cyanobacterial matrix when first collected.

**Fig 7:**
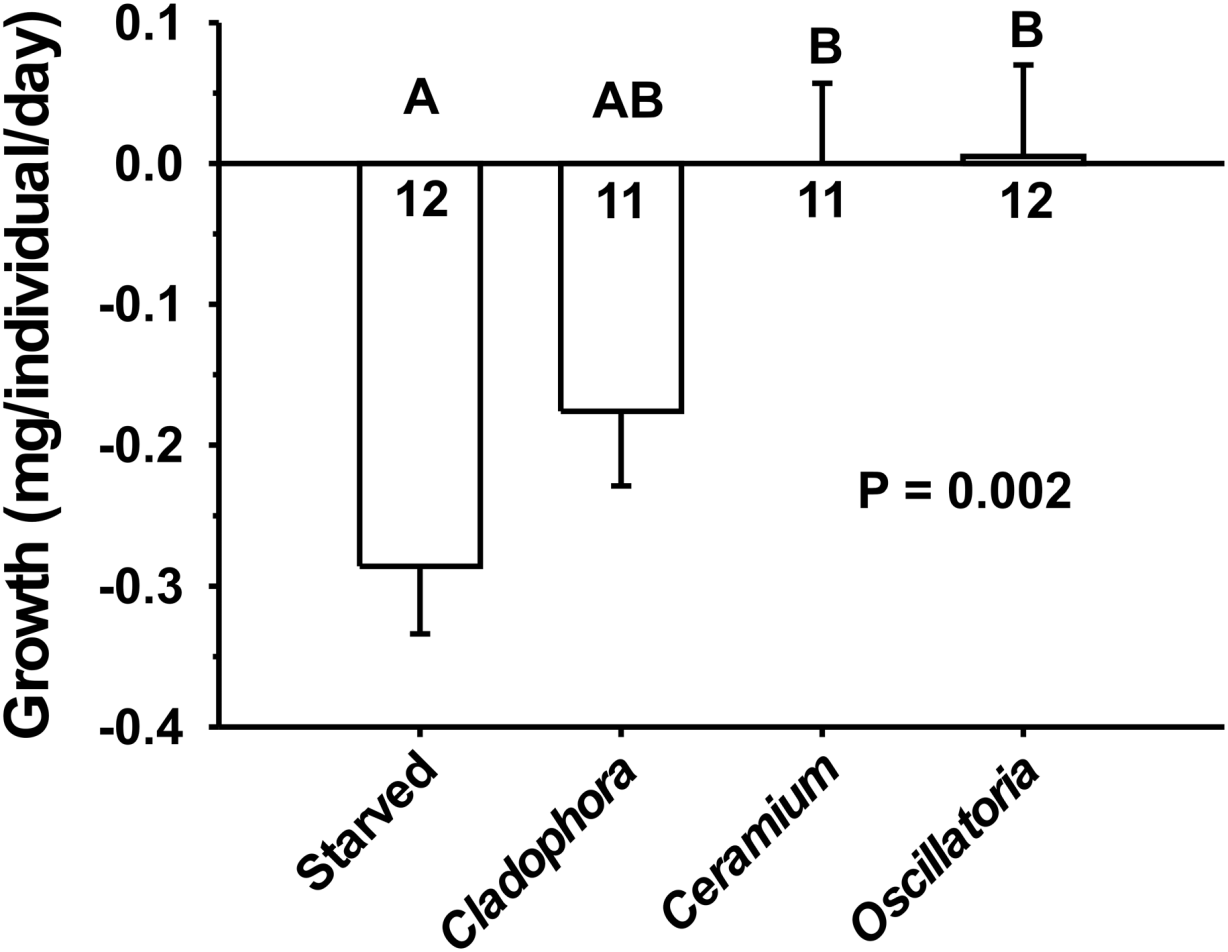
Growth rates of haminoids confined to single diets. Mean growth of *H*. *orbignyana* on two algae, one cyanobacterium, or a starvation control, after 16 days. Symbols and analyses are as in Fig 6. Negative numbers indicate loss of mass.

When raised on algae, cyanobacteria, or starved, cephalaspidean survivorship did not vary significantly among treatments after 16 days (P = 0.757, chi-square analysis). Of the six dead individuals (one starvation control, two fed on *Cladophora*, two fed on *Ceramium*, and one fed on *Oscillatoria*), three died of desiccation after crawling above the water and drying up on the cup lids. In contrast to these patterns, significant differences in growth were found among treatments (P = 0.002, Kruskal-Wallis test; Fig 7). Starved individuals lost significantly more mass than those fed on *Ceramium* or *Oscillatoria*. Animals consuming *Cladophora* lost more mass on average than those in other fed treatments, but these differences were not statistically significant when using the less powerful Tukey-type non-parametric comparisons (Fig 7). Noticeably, snail growth on *Ceramium* and *Oscillatoria* was not significantly different than zero (P = 0.223 and P = 0.679, respectively, Mann-Whitney U tests). Thus, animals in these two diets were capable of maintaining their weight, albeit not grow. It is important to note, though, that it was difficult to maintain the animals fed *ad libitum* on the cyanobacterial food. As explained above, the motility of *Oscillatoria* filaments caused difficulties for harvesting mats from the lab reservoir. Also, while the two algae held well, it was difficult to maintain large amounts of the cyanobacterium in good condition in the laboratory. Thus, the lack of positive growth on this diet should be interpreted with caution.

## Discussion

The unique pear-shaped shell and combination of features of the male reproductive system together with phylogenetic evidence and genetic distances unequivocally demonstrated that the cephalaspidean gastropod that has colonized Lake Qarun is *Haminoea orbignyana*. This species is native to seagrass beds and estuarine habitats in the tropical and temperate NE Atlantic, from France to Nigeria, and the Mediterranean Sea [11, 58, 59]. The impoverished fish and gastropod faunas, along with the historical alteration of chemico-physical parameters in Lake Qarun, have provided *H*. *orbignyana* with a quasi-estuarine environment in which predators and competitors are in low abundance. Salinization of the lake has resulted in the extinction of the original fish species, with the notable exception of the euryhaline *Tilapia zillii*, and it has been noted that most of the nine current fish species in the lake prefer crustacean prey [30, 32, 36]. While some small generalist crabs (*Brachynotus* spp.) share the same microhabitat with the snails, they have occurred at low densities in most (albeit not all) surveys [30]. Furthermore, cephalaspidean gastropods in the genus *Haminoea* (including *H*. *orbignyana*) contain both structural and chemical defenses against predation [60, 61] and the production of chemical deterrents has been linked to the invasion success of the congeneric *H*. *cyanomarginata* from the Red Sea into the Mediterranean [62]. All these factors have likely favored the establishment of this snail species in Lake Qarun and the increase in the population that has been noted in benthic surveys since 1999 [30].

Invasive cephalaspideans have been highlighted previously as examples of the taxonomic impediment. Hanson et al. [8] showed how *Haminoea japonica* had invaded various areas around Europe, sometimes displacing native species almost completely. Yet, this phenomenon had been overlooked until recently because of the lack of experts who can recognize differences between species in the genus (see also [6, 7]). A similar argument can be made for *H*. *orbignyana* from Lake Qarun. Benthic surveys of the lake by El-Shabrawy [45], El-Shabrawy and Dumont [30], and Fouda and Fishar [32] have previously recorded cephalaspidean gastropods. However, these have been identified as either the Indo-Pacific *Bulla ampulla* [63] or *Bulla* sp. It seems unlikely that noticeable numbers of a *Bulla* species would be present in Lake Qarun as recently as 2012 [32] and be completely replaced by *H*. *orbignyana* by 2013, when the animals documented here were collected. Yet, the misidentification is not difficult to understand. Many species in this taxon are small, rare, cryptic, and many remain undescribed [9, 10] while expert taxonomists on the Cephalaspidea are very few worldwide.

Published accounts suggest that *H*. *orbignyana* is a relatively recent introduction to Lake Qarun. Species lists of the molluscs in this lake by Smith [43], Gardner [44], Rose [37], Naguib [36], and Abdel-Malek and Ishak [38], mentioned no Cephalaspidea as components of the benthos. Also consistent with a recent successful introduction, benthic surveys detected an increase in cephalaspideans from 2 individuals/m^2^ to 18 individuals/m^2^ between 1999 and 2006 [30]. Our surveys detected similar densities as those found in 2006 on top of rocks, but we also found 2.7 times as many individuals under rocks (Fig 3). This suggests that these animals could have been introduced considerably earlier than 1999, but their cryptic behavior and putative initial low densities made them difficult to detect. The large numbers of egg masses in our quadrats and co-occurrence of individuals of assorted sizes (suggesting different cohorts) show that these animals are well acclimated to the conditions of the lake, including periods of anoxia and fish die-offs in late summer (Cruz-Rivera, unpublished observations). We know of no previous surveys that have sampled benthic biota under rocks in Lake Qarun, but our data suggest that for certain species, a reassessment of population densities may be warranted. Our surveys were conducted during the day, and the possibility that *H*. *orbignyana* shows diel activity patterns cannot be discarded. Some small opisthobranchs show increased activity at night [64].

While species in the genus *Haminoea* are among the best known examples of bet-hedging through the development of both swimming veligers and crawling larvae [47–49], only veligers were produced in the lab. To our knowledge, no other information on the early ontogeny of *H*. *orbignyana* is available. These results, however, do not conclusively preclude the production of benthic larvae in this species. Although some haminoids produce only veligers [48, 65], for others the fractions of eggs becoming crawling larvae depend on maternal effects, oviposition substrate, and the presence of chemical cues in the egg mass [47, 49]. It is plausible that physico-chemical variables may also affect the production of different larval types, but few studies have addressed this. Our experiments were limited to exposing the egg masses to one set of conditions during development. While temperature, salinity, and pH were very close to average values for the lake [30], all these parameters vary seasonally in Lake Qarun (particularly temperature). The relation between development of *H*. *orbignyana* and water conditions awaits further study.

Malaquias et al. [66] found that gut contents of *H*. *orbignyana* were dominated by diatoms and sand grains. Our specimens lived on a different environment compared to the tidal seagrass beds from that earlier study, so we selected three abundant potential diets to feed and maintain the snails. The haminoid was readily capable of consuming and processing the often abundant algae and cyanobacteria on the rocks it recruits (Figs 4 and 5). Feeding rates were significantly higher on the rhodophyte *Ceramium* than the chlorophyte *Cladophora*, and snail mean growth rates were lower (though not significantly) on the green alga. The difficulty of reliably weighing film-forming cyanobacteria resulted in a large variance in the measured consumption of *Oscillatoria*. However, growth rates showed that snails could at least maintain their mass feeding on either the red alga or the cyanobacterium, compared to starvation controls (Fig 7). While not statistically different to other treatments due to the large variance, animals raised on *Cladophora* lost mass at a rate different than zero, suggesting that—although *H*. *orbignyana* can readily ingest this food (see pictures in Fig 6)–it is not physiologically able to gain enough nutrition from this source. Some marine species of *Haminoea* are known to specialize on cyanobacteria [57], but our difficulties providing snails with sufficient amounts of *Oscillatoria* during the growth assay precluded a clear evaluation of this. Our data do demonstrate that *H*. *orbingyana* can consume and maintain weight on two common primary producers of Lake Qarun. It is possible that these foods could maintain populations in combination, though this has not been tested. It is also possible that populations could be maintained in the absence of a preferred diet on any one of these resources for a limited amount of time.

The problem of salinization in freshwater ecosystems has been highlighted by many researchers [18, 19, 22–25]. Fish biotas, for example, can be highly sensitive to increased salinity, resulting in a displacement of native species and an increase of non-native euryhaline ones [25, 26]. However, a complete shift from a freshwater to a brackish or marine environment, such as the case for Lake Qarun, is seldom observed. Such examples (e.g., Garaet El Ichkeul in Tunisia, Sabaudia Lake in Italy) should, nevertheless, be observed as plausible scenarios if proper management is overlooked [21, 22, 26, 29]. Salinity in Lake Qarun rose from ca. 10 to 45 psu, in less than a century and it currently stands at about 32 psu, with some seasonal fluctuations [29, 30]. Thus, environmental changes have favored the establishment of a marine invertebrate fauna in a matter of decades, from an original freshwater assemblage that lasted thousands of years [28, 30, 31, 37, 44]. It is relevant to note that salinity increases in Sabaudia Lake, Italy, have been related to the temporary colonization (1974–1995) of that lake also by *H*. *orbignyana* [22], but the species now appears to have been replaced by the congeneric and invasive *H*. *japonica* [7, 8, 22].

While the ultimate origin of these lake cephalaspideans cannot be known without further study, it is possible that their introduction is related to fisheries activities in Lake Qarun. Fishes and prawns have been brought from the Mediterranean coast of Egypt to seed the lake since the late 1920s [32, 39–42]. The lake is very shallow and landlocked (Fig 1), so boat traffic from the sea or ballast water are unlikely transport routes. The regular stocking of the lake with Mediterranean species provides a clear potential route for the introduction of *H*. *orbignyana*. It is interesting to note that records of new molluscs of Mediterranean origin in Lake Qarun have been published before, but these instances have been interpreted as random natural phenomena. For example, Rose [37] discovered that the brackish water Mediterranean snail *Pirenella conica* dominated the southern shores of Lake Qarun. He argued that this, and other Mediterranean molluscan species in the lake, must have been introduced by birds. While *P*. *conica* still inhabits the lake [30, 32], no specimens were observed during our surveys. Clearly, phoretic transportation of species is a possibility in a lake that is used by a variety of migratory bird species [30]. However, cephalaspideans lack the hard protective shells and sealing opercula that snails like *P*. *conica* have, making *H*. *orbignyana* more sensitive to desiccation and breakage, and this route of introduction less plausible.

## Conclusions

This work conclusively identifies the first species of a cephalaspidean mollusc recorded from a completely closed lake. Central to the successful range shift for this species is the progressive change of physico-chemical parameters that Lake Qarun has endured as salinization has increased in the past century. The clearly dynamic nature of this system precludes any robust predictions regarding the long term fate or impact of *H*. *orbignyana* populations on the benthos, but under the current conditions of Lake Qarun, this introduced species is thriving.

## Supporting Information

S1 Table *Haminoea* individuals and egg mass abundance. (XLSX)

S2 Table Feeding of *Haminoea* in no-choice experiment. (XLSX)

S3 Table Growth of *Haminoea* over 16 days. (XLSX)
